# 2D-gel Electrophoresis As a Tool to Investigate the Composition of CD95 DISC

**Published:** 2010-07

**Authors:** D. Riess, I. Lavrik

**Affiliations:** Division of Immunogenetics, German Cancer Center, Heidelberg, Germany

**Keywords:** apoptosis, receptor CD95, 2D-gel electrophoresis

## Abstract

Stimulation of CD95 (APO–1/Fas) leads to apoptosis induction in multicellular organisms. CD95–mediated apoptosis starts with the formation of the protein complex at the receptor CD95 (APO–1/Fas), which was named DISC (death–inducing signaling complex). In this work, the composition of the CD95 DISC in two different cell types was analyzed using proteomics approaches. Using 2D gels, the composition of the CD95 DISC was analyzed in the so–called Type I and Type II cells, which are characterized by different kinetics of apoptosis. The detailed analysis of the CD95 DISC performed by 2D gels demonstrated that, besides the well–established components of the CD95 DISC, which are present in both cell types (CD95, FADD and procaspase–8), there are a number of differential spots detected at the CD95 DISC of Type I * versus * Type II cells. Taken together, this work demonstrates the differential composition of the CD95 DISC of Type I * versus* Type II cells.

## INTRODUCTION

Apoptotic cell death is common to multicellular organisms and can be triggered by a number of factors, including UV– or γ –irradiation, chemotherapeutic drugs, growth factor withdrawal, and signaling from death receptors (1, 2).

The death receptor family comprises the following receptors: TNF–R1, CD95 (APO–1/Fas), DR3, TRAIL–R1, TRAIL–R2, DR6, EDA–R, and NGF–R (2). It is considered that, for efficient signal transduction, death receptors have to form oligomers, probably trimers (1–3). The cytoplasmic part of the death receptors contains the so–called death domains (DD), which play the central role in the transduction of the apoptotic signal. DDs can undergo homotypic oligomerization with other molecules containing DD. In this way, adaptor molecules can bind to the receptors, forming a receptor–signaling complex.

**Fig. 1:**
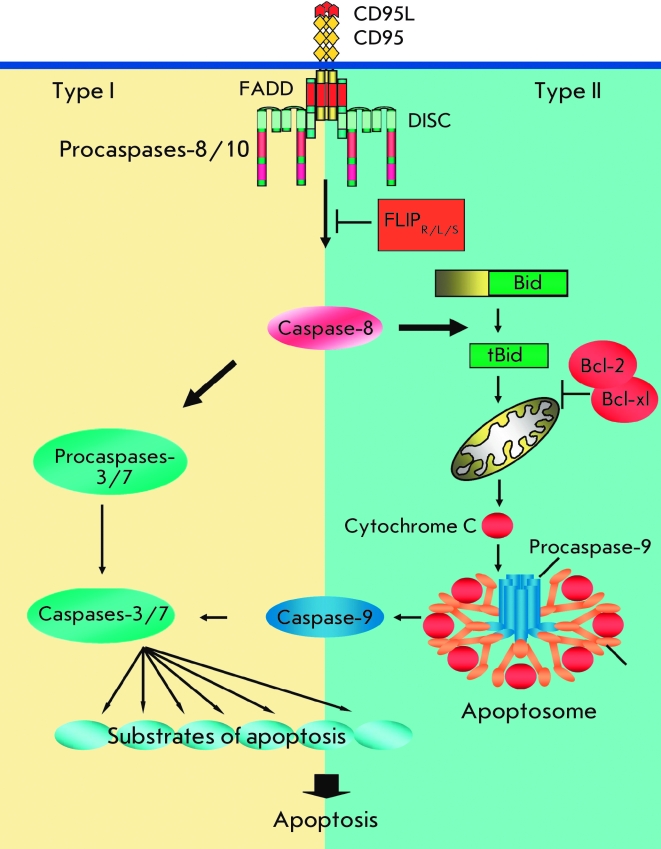
Figure 1. Scheme of Type I and Type II apoptotic pathways. In the CD95 pathway, stimulation with CD95L of CD95 leads to formation of the death-inducing signaling complex (DISC), where activation of procaspase-8 and procaspase-10 takes place. Caspase-8-mediated apoptosis occurs in different ways in type I vs. type II cells. Type I cells are characterized by high levels of DISC formation and increased amounts of active caspase-8 (left-hand side). Caspase-8 cleaves and thereby activates the downstream effector caspase-3, caspase-6, and caspase-7. In type II cells, there are lower levels of CD95 DISC formation and, therefore, lower levels of active caspase-8 (right-hand side). In this case, signaling requires an amplification loop that involves the cleavage by caspase-8 of the Bcl-2-family protein Bid to generate truncated (t) Bid and a subsequent tBid-mediated release of cytochrome C (cyt C) from mitochondria. The release of cyt C from mitochondria results in apoptosome formation, followed by the activation of initiator procaspase-9, which in turn cleaves downstream effector caspases. Type II CD95 signaling might be blocked by Bcl-2 family members such as Bcl-2 and Bcl-x_L_. Activation of procaspase-8 at the DISC can be blocked by c-FLIP proteins.

CD95/APO–1/Fas–mediated apoptosis is one of the most studied apoptotic signaling pathways (1, 3). The CD95 DISC formation occurs within seconds after the binding of CD95L to CD95 (3–5). All interactions at the CD95 DISC are based on homotypic interactions. First, FADD (Fas–associated DD) binds to the DISC * via * DD interactions. The FADD molecule also contains DED (Death effector domain), which allows recruitment of procaspase–8 into the receptor complex * via * DED interactions. Procaspase–8 undergoes autocalytic activation at the DISC with the generation of the active form of caspase–8 (Fig. 1). This results in the activation of the effector caspases–3 and –7, which is followed by the cleavage of the apoptotic substrates, leading to cell death (2) (Fig. 1).

Two CD95 signaling pathways have been identified so far (4) (Fig. 1). Type I cells are characterized by high levels of CD95 DISC formation and increased amounts of active caspase–8, which activates downstream effector caspases–3 and –7. Type II cells are characterized by lower levels of CD95 DISC formation and, thus, lower levels of active caspase–8. In this case, signaling requires an additional amplification loop that involves the cleavage of the Bcl–2–family protein Bid by caspase–8 to generate truncated (t)Bid and subsequent (t)Bid–mediated release of cytochrome C from mitochondria. The release of cytochrome C from mitochondria results in apoptosome formation, followed by activation of procaspase–9, which in turn cleaves downstream effector caspases. Among T and B cell lines, Type I cells comprise: B cell lines SKW6.4, Raji, BJABs and T cell line Hut78, as well as peripheral T cells. It has been shown that Type II cells comprise T cell lines CEM and Jurkat (4).

The nature of the different kinetics of caspase–8 activation at the DISC in Type I and Type II cells is not established yet and, probably, might be due to the different protein composition of the CD95 DISC of Type I * versus * Type II cells. The goal of this study was to verify this hypothesis and to compare the protein pattern of the CD95 DISC of Type I cells * versus * Type II cells with proteomics approaches using 2D gels.

2D gels are based on the separation of proteins in the first direction based on their isoelectric point (pI), which is followed by the separation of proteins in the second direction based on their molecular mass (М_r_) (6). This approach plays a very important role in proteomics studies. The 2D gels approach is also applied with different modifications, which allows to analyze protein complexes of different complexities. We used the approach with immobilized pH–gradients (IPG), which has been shown to possess high reproducibility (7). In this work, we have analyzed the composition of the CD95 DISC in a pI interval ranging from 3 to 10 and developed conditions for 2D gels in a pI range from 6 to 11. Our work has shown different protein compositions of the CD95 DISC immunoprecipitated from Type I * versus * Type II cells.

## MATERIALS AND METHODS

**Cell lines **. The B lymphoblastoid cell line SKW6.4 and the T cell line CEM were maintained in RPMI 1640 (Life Technologies, Germany), 10 mM HEPES (Life Technologies, Germany), 50 µg/ml Gentamycin (Life Technologies, Germany), and 10% fetal calf serum (Life Technologies, Germany) in 5% CO_2_.

**Antibodies and reagents **. Anti–CD95 polyclonal antibodies C20 were purchased from Santa Cruz Biotechnology (Heidelberg, Germany). CD95L was prepared as described in (8). The anti–FADD mAb 1C4 (mouse IgG1) recognizes the C–terminus of FADD. The anti–caspase–8 mAb C15 and mAb C5 (mouse IgG2b and IgG2a, respectively) recognize the p18 subunit of caspase–8 and the p10 subunit of caspase–8 (9). Anti–APO–1 is an agonistic monoclonal antibody recognizing an epitope on the extracellular part of CD95 (APO–1/Fas) (10). Horseradish peroxidase–conjugated goat anti–mouse IgG1, –2a, and –2b were from Southern Biotechnology Associates (United Kingdom). [^35^S]Met and [^35^S]Сys were purchased from Amersham. All the other chemicals used were of analytical grade and purchased from Merck (Germany) or Sigma (Germany).

**Preparation of total cellular lysates **. ** 1 x 10^8^ cells were washed twice in 1 x PBS and subsequently lysed in buffer A (20 mM Tris/HCl, pH 7.5, 137 mM NaCl, 2 mM EDTA, 1 mM phenylmethylsulfonyl fluoride (Sigma, Germany), protease inhibitor cocktail (Roche, Switzerland), 1% Triton X–100 (Serva, Germany) and 10% glycerol) (stimulation condition) or lysed without treatment (unstimulated). The total cellular lysates were subsequently analyzed by Western Blot.

**DISC analysis by immunoprecipitation and Western Blot **. 5 x 10^7^ SKW6.4 cells or 7 x 10^7^ CEM cells were treated with 1 µg/ml of LZ–CD95L at 37°C for the indicated periods of time, washed twice in 1 x PBS, and subsequently lysed in buffer A (stimulation condition) or lysed without treatment (unstimulated). The CD95 DISC was immunoprecipitated overnight with 2 µg of anti–APO–1 and protein A sepharose beads (11). Protein A sepharose beads were washed five times with 20 volumes of lysis buffer. The immunoprecipitates were analyzed on the 12% PAGE. Subsequently, the gels were transferred to the Hybond nitrocellulose membrane (Amersham Pharmacia Biotech., Germany), blocked with 5% nonfat dry milk in PBS/Tween (PBS plus 0.05% Tween 20) for 1 h, washed with PBS/Tween, and incubated with the primary antibodies in PBS/Tween at 4°C overnight. Blots were developed with a chemoluminescence method following the manufacturer’s protocol (Perkin Elmer Life Sciences, Germany). 

**Labeling of the cells with [** *^35^* *S* **]**

5 х 10^7^ SKW6.4 cells or 7 х 10^7^ CEM cells were incubated for one hour at 37^0^С in RPMI media without methionine and cysteine. Afterwards, [^35^S]Met and [^35^S]Сys were added to the cells, and cells were cultured 24 h before performing the experiments. 

**2D–gels **. To perform isoelectrofocusing (IEF) **** of the lysates, **** 10 µ λ of total cellular lysates were added to 340 µ λ of the buffer B (9М urea, 2% СНАPS, 18 mМ DTT, 0.001% bromphenol blue, 0.5 % IPG buffer (Amersham)) or buffer C (9М urea, 2% NP–40, 18 mМ DTT, 0.001% bromphenol blue, 0.5 % IPG buffer (Amersham)).

To perform isoelectrofocusing (IEF) **** of the immunoprecipitates, proteins after immunoprecipitation were eluted from protein A sepharose beads for 30 minutes at room temperature using buffer B.

After isoelectrofocusing, IPG stripes were equilibrated for 20 minutes in the buffer D: 50 mМ tris–HCl, pH 8.8,6 M urea, 30% glycerol, 65 mМ DTT, 0.001% bromphenol blue, which was followed with the incubation in buffer D containing 2.5% of iodoacetamide. Afterwards, IPG stripes were fixed with 0.5% agarose at 12% SDS–PAGE, which was followed by electrophoresis in the second direction. Afterwards, gels were analyzed using autoradiography or Western Blot. In some cases, the gels were stained using the SiverQuest Silver Staining Kit from Invitrogen.

## RESULTS AND DISCUSSION

**Analysis of the CD95 DISC using 2D gels at a pI range from 3 to 10 **. To undertake the proteomics analysis of the CD95 DISC composition, we selected two cell lines: B lymphoblastoid cells SKW6.4 as Type I cells and T cell line CEM as Type II cells. Both cell lines were characterized in detail in previous works and were demonstrated to possess typical features of Type I (SKW6.4 cells) * versus * Type II cells (CEM cells) (4). 

 To analyze the CD95 DISC composition, SKW6.4 and CEM cells were first cultured with [^35^S]Met and [^35^S]Сys for one day. Afterwards, cells were stimulated with LZ–CD95L. CD95 DISC was immunoprecipitated using monoclonal antibodies anti–APO–1. Anti–APO–1 antibodies recognize the extracellular domain of CD95 and have been used for the CD95 DISC immunoprecipitation in previous works (10, 11, 12). Immunoprecipitates were analyzed using 2D gels.

**Fig. 2:**
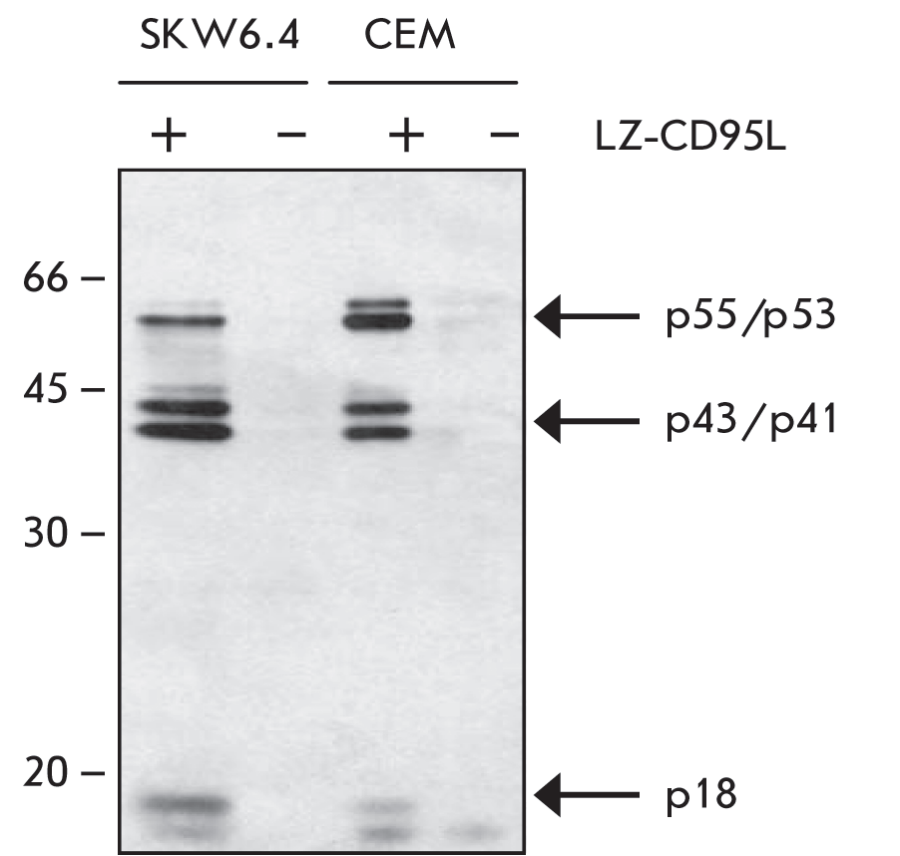
Figure 2. Control of the CD95 DISC formation using Western Blot. CD95 DISC formation (1/10 of the amount of protein loaded on 2D gels) was analyzed using 1D gels with subsequent Western Blot analysis using monoclonal antibodies C15 against caspase-8. The position of procaspase-8 (р55/р53) and its cleavage products р43/р41, р18, and р10 is indicated.

To control the immunoprecipitation, a tenth of the sample which was used for 2D gels was loaded onto the 1D gels and controlled using Western Blot and specific antibodies against procaspase–8 (Fig. 2). This analysis demonstrated the presence of procaspase–8, as well as its cleavage products p43/p41 at the DISC, showing the specificity of the methods used. 

**Fig. 3:**
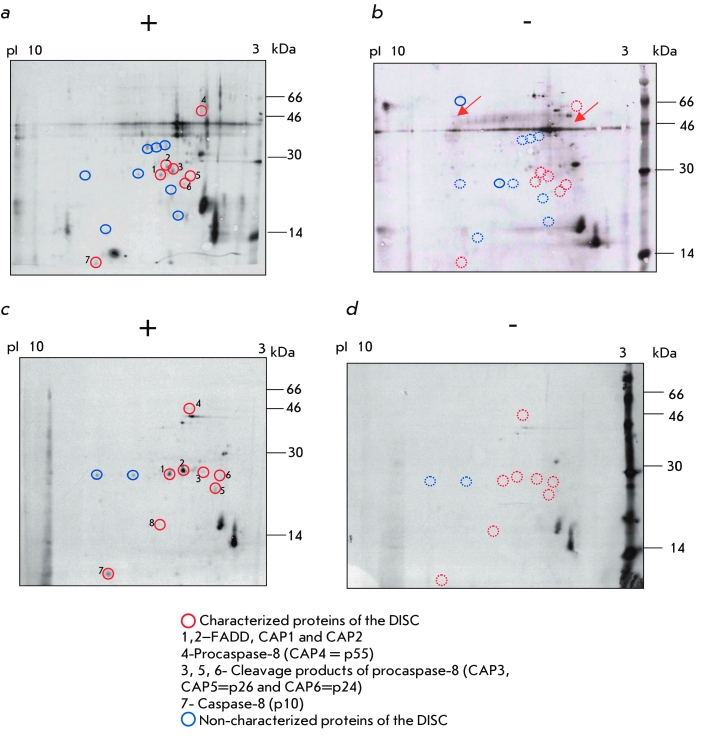
Figure 3. The analysis of the CD95 DISC in Type I cells (SKW6.4) and Type II cells. 5 х 10^7^ of SKW6.4 cells were stimulated with LZ-CD95L (а) or left untreated (b). 7 х 10^7^ of СЕМ cells were stimulated with LZ-CD95L (c) or left untreated (d). CD95 DISC immunoprecipitations were analyzed using 2D gels in the pI range from 3 to 10. The autoradiograms were exposed for 4 weeks. One out of three representative experiments is shown. CD95 position is indicated. All main components of the CD95 DISC are indicated with numbers. Differential spots are shown with a solid line. The disappearance of the spot is shown with a dashed line.

 The composition of the CD95 DISC after immunoprecipitation was first analyzed using 2D gels with a pI range varying from 3 to 10 (Fig. 3). The 2D gel analysis of the CD95 DISCs of Type I * versus * Type II cells revealed the presence of spots with a pI and molecular mass corresponding to the main proteins of the CD95 DISC described in previous works (5, 13). The following proteins were detected: CD95, FADD, which is present in two forms: CAP1 (cytotoxicity–associated protein 1) and CAP2 (non–phosphorylated and phosphorylated FADD, respectively), procaspase–8 (CAP4) and its cleavage products САР3, р26/р24, р18, and р10. In the DISC of both Type I and Type II cells, new non–characterized proteins were detected, as can be seen at corresponding 2D gels (Fig. 3). Interestingly, the molecular masses and pI of the new proteins of the CD95 DISC were different in Type I * versus * Type II cells, which indicates the differential composition of the CD95 DISC in these two cell types.

**Analysis of the CD95 DISC using 2D gels at a pI range from 6 to 11 **. Since the resolution of the 2D gels at the region above the pI is relatively low, the proteins with basic pIs might not be detected using this approach. Therefore, we decided to analyze the protein composition of the DISC at a pI range varying from 6 to 11.

**Fig. 4:**
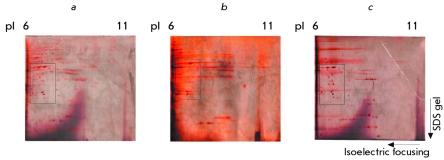
Figure 4. Optimization of isoelectrofocusing conditions. We performed isoelectrofocusing of the cellular lysates obtained from 107 of SKW6.4 cells. Afterwards, the samples were loaded on a 12 % PAGE. The proteins were detected using silver stain. The composition for the buffers for electrofocusing was as follows: (а, c) 9 М urea, 2 % СНАPS, 18 mМ DTT, 0.001 % bromphenol blue, and 0.5 % of IPG buffer from Amersham. (b) 9 М urea, 2 % NP-40, 18 mМ DTT, 0.001 % bromphenol blue, and 0.5 % of IPG buffer from Amersham. Isoelectrofocusing has been performed: (а, b) 12 h - dehydration, 1 h-500 V, 1 h-1000 V, 1 h- 3000 V, 8000 V until 60000 Vh. (c) 12 h- dehydration, 1 h -100 V, 1 h – 150 V, 1 h – 300 V, 1 h – 400 V, 1 h – 500 V, 1 h – 1000 V, 1 h – 3000 V, 8000 V to until 180000 Vh.

It was shown that isoelectrofocusing at a pI range varying from 6 to 11 is complicated due to the electroosmotic flow of water and migration of the DTT in the direction of the anode (14). This makes it difficult to obtain 2D gels of high quality at a pI range varying from 6 to 11. Therefore, first we had to optimize the conditions of isoelectrofocusing in a pI range from 6 to 11. First, lysates of SKW6.4 cells were prepared, and isoelectrofocusing was carried out using different detergents: e.g. CHAPS and NP–40. In addition, different protocols for isoelectrofocusing were utilized (Fig. 4). The quality of the 2D gels was judged according to the number of spots and their resolution.

The detergent CHAPS (Fig. 4A) provided a much better resolution in comparison with the detergent NP–40 (Fig. 4B). Isoelectrofocusing which was performed in experiments presented in Figs. 4A and 4B comprised four regimes: 500V, 1000V, 3000V, and 8000V. To improve the quality of the 2D gels, we also applied conditions for isoelectrofocusing with eight regimes of focusing from 100V up to 8000V and a longer time of isoelectrofocusing (Fig. 4C). Apparently, these conditions resulted in a much better resolution of the 2D gels (Fig. 4C). Therefore, for the next experiments we used detergent CHAPS and conditions for isoelectrofocusing as shown in Fig. 4C.

For the comparative analysis of the CD95 DISC of Type I * versus* Type II cells in this pI range, we also selected SKW6.4 cells as Type I cells and CEM cells as Type II cells. Experiments were performed similarly as described above for the analysis for a pI varying from 3 to 10. The SKW6.4 and CEM cells were cultured with [^35^S]Met and [^35^S]Сys for 24 hours. To induce the formation of the CD95 DISC, cell cultures were treated with LZ–CD95L. This was followed by the immunoprecipitation of the CD95 DISC using monoclonal antibodies anti–APO–1 and 2D gel analysis at a pI range from 6 to 11.

**Fig. 5:**
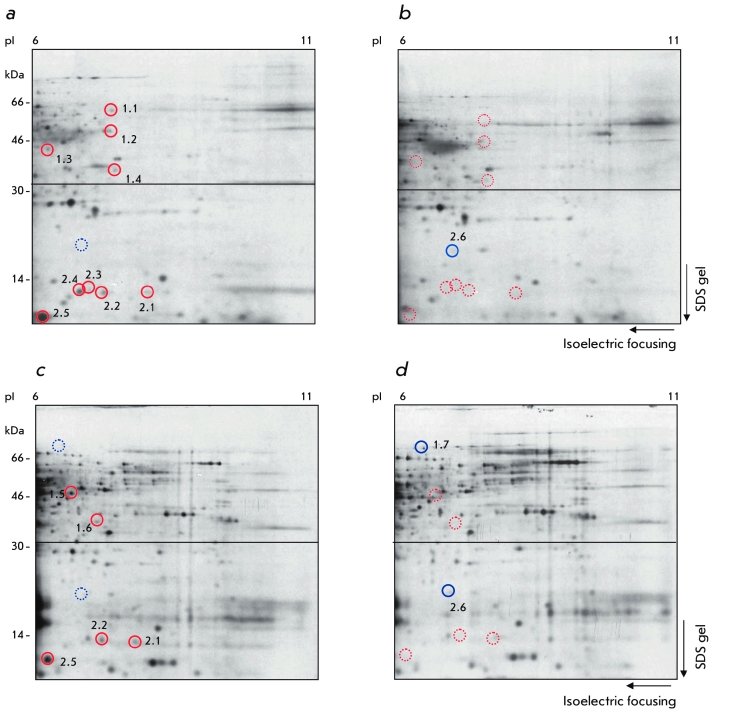
Figure 5. Analysis of the CD95 DISC in Type I cells (SKW6.4) and Type II cells (CЕМ). 5 х 10^7^ of SKW6.4 cells were stimulated with LZ-CD95L (a) or left untreated (b). 7 х 10^7^ of СЕМ cells were stimulated with LZ-CD95L (c) or left untreated (d). CD95-DISC was analyzed using 2D gels in the pI range from 6 to 11. The autoradiograms were exposed for 4 weeks. One out of three representative experiments is shown. Differential spots are shown with a solid line. The disappearance of the spot is shown with a dashed line.

The analysis of 2D gels revealed a number of new proteins present in the CD95 DISC of Type I cells (Fig. 5A), as well as in Type II cells (Fig. 5B). All proteins with a molecular mass of more than 30 kDa are identified with numbers from 1.1 to 1.7. All proteins with a molecular mass lower than 30 kDa are identified with numbers from 2.1 tо 2.7 (Fig. 5). The molecular masses and pIs of the new proteins of the CD95 DISC of Type I and Type II cells were different, with the exception of the spot 2.5, which was observed in both cell types. Therefore, we were able to demonstrate that the protein composition of the CD95 DISC in Type I cells is different from the CD95 DISC in Type II cells also in a pI range from 6 to 11.

**Fig. 6:**
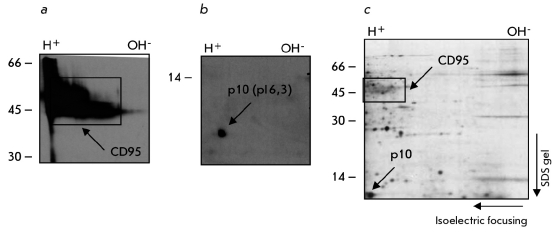
Figure 6. Analysis of the 2D gels using Western Blot. CD95 DISC, immunoprecipitated from SKW6.4, was analyzed using 2D gels with Western Blot and specific antibodies C20 against CD95 (а), specific antibodies C15 against caspase-8 (b). The position of caspase-8 and CD95 is shown at autoradiogram (c).

To control the immunoprecipitation, a tenth of the CD95 DISC immunoprecipitation was loaded on the 1D gel. This was followed by Western Blot with the specific antibodies against established components of the CD95 DISC as was demonstrated in Fig. 2. In addition, we analyzed 2D gels using Western Blot and we detected the presence of already known components of the CD95 DISC: CD95 (Fig. 6A, B) and active caspase–8, р10 (Fig. 6C). The comparison of the pI and molecular mass of the active caspase–8 at the Western Blot with spots at autoradiogramms showed that the protein 2.5 corresponds to the active caspase–8 (Fig. 6D). Thus, we, for the first time, had established the conditions for 2D gels in a pI range varying from 6 to 11 needed to analyze the proteins associated with CD95.

## CONCLUSIONS

Therefore, the application of 2D gel electrophoresis has allowed us to analyze the composition of the CD95 DISC in Type I * vs. * Type II cells. Notably, 2D gel analysis has revealed the differential spots at the 2D gel of Type I * vs. * Type II cells, which confirms the hypothesis that the differential kinetics of caspase–8 activation in Type I * vs. * Type II cells is based on the different protein compositions of the CD95 DISC. At the moment, we are attempting to identify new proteins using mass–spectrometry analysis.
